# CYP19A1 regulates chemoresistance in colorectal cancer through modulation of estrogen biosynthesis and mitochondrial function

**DOI:** 10.1186/s40170-024-00360-4

**Published:** 2024-10-28

**Authors:** Yang Wang, Qiang Ji, Ning Cao, Guijie Ge, Xiaomin Li, Xiangdong Liu, Yanqi Mi

**Affiliations:** 1 Department of General Surgery, Longhua Hospital, Shanghai University of Traditional Chinese Medicine, Shanghai, China; 2 Department of Pharmacy, Sunshine Union Hospital, Weifang, Shandong China; 3 Emergency General Surgery, Weifang People’s Hospital, Shandong Second Medical University, Weifang, Shandong China; 4 Department of Anesthesiology II Endoscopy Center, Weifang People’s Hospital, Weifang, Shandong China; 5 Medical Center of gastrointestinal Surgery, Weifang People’s Hospital, Weifang, Shandong China; 6 Department of Pharmacy, Weifang People’s Hospital, Weifang, Shandong China

**Keywords:** Colorectal cancer, Chemotherapy resistance, CYP19A1, Mitochondria, Mitochondrial respiratory chain complexes

## Abstract

Chemoresistance remains a major challenge in the effective treatment of colorectal cancer (CRC), contributing to poor patient outcomes. While the molecular mechanisms underlying chemoresistance are complex and multifaceted, emerging evidence suggests that altered mitochondrial function and hormone signaling play crucial roles. In this study, we investigated the role of CYP19A1, a key enzyme in estrogen biosynthesis, in regulating chemoresistance in CRC. Using a combination of in vitro functional assays, transcriptomic analysis, and clinical data mining, we demonstrate that CYP19A1 expression is significantly upregulated in CRC cells and patient-derived samples compared to normal controls. Mechanistically, we found that CYP19A1 regulates chemoresistance through modulation of mitochondrial function and complex I activity, which is mediated by CYP19A1-dependent estrogen biosynthesis. Notably, targeted inhibition of CYP19A1 and complex I using specific inhibitors effectively reversed the chemoresistance of CRC cells to chemotherapeutic drugs. Moreover, analysis of the TCGA CRC dataset revealed that high CYP19A1 expression correlates with poor overall survival in chemotherapy-treated patients. Taken together, our findings uncover a novel role for CYP19A1 in regulating chemoresistance in CRC through modulation of mitochondrial function and estrogen signaling, and highlight the potential of targeting the CYP19A1/estrogen/complex I axis as a therapeutic strategy to overcome chemoresistance and improve patient outcomes.

**Supplementary Information:**

The online version contains supplementary material available at 10.1186/s40170-024-00360-4.

## Introduction

Colorectal cancer (CRC) is a leading cause of cancer-related mortality worldwide [1, 2]. Despite significant advances in early detection and treatment strategies, chemoresistance remains a major obstacle to effective CRC management, contributing to poor patient prognosis and high mortality rates [3–5]. Therefore, elucidating the molecular mechanisms underlying chemoresistance and identifying novel therapeutic targets are crucial for improving CRC treatment outcomes and patient survival.

Mitochondria play a central role in cellular energy metabolism, apoptosis, and redox homeostasis, making them key players in cancer development and progression [6–8]. Accumulating evidence suggests that mitochondrial function and metabolism play a critical role in the development of chemoresistance in various cancers, including CRC [9–11]. In particular, alterations in mitochondrial respiration have been implicated in the modulation of drug response and the acquisition of resistant phenotypes [12, 13]. However, the precise molecular mechanisms by which mitochondrial function influences chemoresistance in CRC remain poorly understood.

Cytochrome P450 family 19 subfamily A member 1 (CYP19A1), also known as aromatase, is a key enzyme responsible for the biosynthesis of estrogens from androgenic precursors [14]. While the role of CYP19A1 in hormone-dependent cancers has been extensively studied [15–18], its function in CRC remains largely unexplored. Given the importance of estrogen signaling in various cellular processes, including mitochondrial function and energy metabolism [19–21], we hypothesized that CYP19A1 might play a role in regulating mitochondrial activity and chemoresistance in CRC.

In this study, we investigated the role of CYP19A1 in modulating chemoresistance in CRC cells and explored the underlying molecular mechanisms. We demonstrated that CYP19A1 reverses chemoresistance by regulating mitochondrial function and complex I activity through a CYP19A1/estrogen/complex I signaling axis. Furthermore, we showed that targeting CYP19A1 or complex I with specific inhibitors effectively sensitizes chemoresistant CRC cells to chemotherapy. Finally, using clinical data from The Cancer Genome Atlas (TCGA) database, we revealed a significant correlation between CYP19A1 expression and overall survival in chemotherapy-treated CRC patients. Our findings provide novel insights into the molecular basis of chemoresistance in CRC and highlight the CYP19A1/estrogen/complex I axis as a promising therapeutic target for overcoming drug resistance and improving patient outcomes.

## Materials and methods

### Cell culture

Human CRC cell lines SW480 and HT29 were obtained from the American Type Culture Collection (ATCC). Both cell lines were cultured in Dulbecco’s Modified Eagle Medium (DMEM) supplemented with 10% fetal bovine serum (FBS), 100 U/mL penicillin, and 100 µg/mL streptomycin at 37 °C in a humidified atmosphere containing 5% CO2.

## TCGA data analysis

RNA-seq data and clinical information for CRC patients were obtained from The Cancer Genome Atlas (TCGA) database colon adenocarcinoma (TCGA-COAD) dataset using the TCGAbiolinks R package. CYP19A1 expression levels were extracted as Transcripts Per Million (TPM) values. To compare CYP19A1 expression levels between CRC and normal samples, boxplots were generated using the ggplot2 R package. The Wilcoxon rank-sum test was applied to determine the statistical significance of expression differences between tumor and normal tissues. Patients were stratified into high and low CYP19A1 expression groups based on the median expression value. Kaplan-Meier survival analysis was performed using the survival R package. Log-rank tests were used to assess statistical significance of survival differences between groups. For multivariate Cox regression analyses, we utilized the ‘coxph’ function from the survival package in R. We constructed models incorporating CYP19A1 expression levels, sex, and their interaction term. Separate models were built for each treatment regimen (5FU, OXA, and No treatment). Hazard ratios, 95% confidence intervals, and p-values were extracted using the ‘summary’ function. Forest plots visualizing the Cox regression results were created using the ggplot2 package in R.

## Real-time PCR

Total RNA was isolated using the RNeasy Mini Kit (Qiagen) and reverse-transcribed into cDNA using the High-Capacity cDNA Reverse Transcription Kit (Applied Biosystems). Real-time PCR was performed using the PowerUp SYBR Green Master Mix (Applied Biosystems) on a QuantStudio 6 Flex Real-Time PCR System (Applied Biosystems). The relative expression of CYP19A1 was normalized to the housekeeping gene TUBA1A and calculated using the 2-ΔΔCt method. The following primers were used:

CYP19A1_forward: 5’-GACGTCGCGACTCTAAATTGC-3’,

CYP19A1_reverse: 5’-GCACGATGCTGGTGATGTTA-3’,

TUBA1A_forward: 5’-GAAGCAGCAACCATGCGTGA-3’,

TUBA1A_reverse: 5’-CCCCCAATGGTCTTGTCACT-3’.

## Western blot analysis

Cells were lysed in RIPA buffer (50 mM Tris-HCl pH 8.0, 150 mM NaCl, 1% NP-40, 0.5% sodium deoxycholate, 0.1% SDS) supplemented with protease and phosphatase inhibitors. Protein concentrations were determined using the BCA Protein Assay Kit (Thermo Fisher Scientific). Equal amounts of protein were separated by SDS-PAGE and transferred to PVDF membranes. Membranes were blocked with 5% non-fat milk and incubated with primary antibodies against CYP19A1 (cat #: HPA051194, Sigma, 1:1000) and TUBA1A (cat #: ab95966, Abcam, 1:5000) overnight at 4 °C. After incubation with HRP-conjugated secondary antibodies. The signal was detected using the ECL substrate (Thermo Fisher Scientific).

## Immunofluorescence staining

Cells were seeded on glass coverslips and allowed to attach. For the mitochondrial localization study, cells were first incubated with 500 nM MitoTracker Red CMXRos (Invitrogen) in serum-free medium for 30 min at 37 °C, then washed with PBS. For both studies, cells were fixed with 4% paraformaldehyde, permeabilized with 0.1% Triton X-100, and blocked with 5% BSA. Cells were then incubated overnight at 4 °C with primary antibodies: anti-CYP19A1 (cat #: HPA051194, Sigma, 1:100 dilution) for both studies, and additionally anti-calreticulin (cat #: MA5-45067, Invitrogen, 1:100 dilution) for the endoplasmic reticulum localization study. Following primary antibody incubation, cells were treated with appropriate Alexa Fluor-conjugated secondary antibodies (Invitrogen) for 1 h at room temperature: Alexa Fluor 488 for CYP19A1 in both studies, and Alexa Fluor 594 for calreticulin in the endoplasmic reticulum study. Nuclei were counterstained with DAPI. Images were acquired using a confocal microscope (Zeiss LSM 800).

### Generation of CYP19A1 knockout cell lines using CRISPR-Cas9

CYP19A1 knockout SW480 and HT29 cell lines were generated using the CRISPR-Cas9 system. Two guide RNAs (gRNAs) targeting exon 3 (TCTCCCACGGCAGATTCCTG) and exon 5 (AGACTGTGACCATACGAACA) of the CYP19A1 gene were cloned into the pSpCas9(BB)-2 A-Puro (PX459) V2.0 vector (Addgene, #62988). Cells were co-transfected with both gRNA-containing plasmids using Lipofectamine 3000 (Invitrogen) according to the manufacturer’s instructions. After 48 h, transfected cells were selected with 2 µg/mL puromycin for 7 days. Single-cell clones were isolated by limiting dilution and expanded.

## Seahorse mito stress test

Oxygen consumption rate (OCR) was measured using the Seahorse XFe96 Extracellular Flux Analyzer (Agilent) according to the manufacturer’s instructions. Cells were seeded in XFe96 cell culture microplates at a density of 20,000 cells per well and allowed to attach overnight. Prior to the assay, the growth medium was replaced with Seahorse XF Base Medium supplemented with 10 mM glucose, 1 mM pyruvate, and 2 mM glutamine, and cells were incubated at 37 °C in a non-CO2 incubator for 1 h. OCR was measured at baseline and after sequential injection of oligomycin (1 µM), FCCP (0.5 µM), and rotenone/antimycin A (0.5 µM) to assess basal respiration, ATP-linked respiration, and maximal respiration. Basal respiration was calculated as the difference between the baseline OCR and the OCR after rotenone/antimycin A injection. ATP-linked respiration was calculated as the difference between the baseline OCR and the OCR after oligomycin injection. Maximal respiration was calculated as the difference between the OCR after FCCP injection and the OCR after rotenone/antimycin A injection. After the assay, cells were lysed, and the protein concentration in each well was determined using the BCA Protein Assay Kit (Thermo Fisher Scientific). All OCR values were normalized to the protein concentration in each well to account for potential differences in cell number.

## Assessment of mitochondrial function, cellular metabolism, and estrogen levels

Mitochondrial complex I, III, IV, and V activities were measured using commercially available assay kits from Abcam. Complex I activity was determined using the Complex I Enzyme Activity Microplate Assay Kit (ab109721). Complex III activity was assessed with the Complex III Enzyme Activity Microplate Assay Kit (ab287844). Complex IV activity was measured using the Complex IV Human Enzyme Activity Microplate Assay Kit (ab109909). Complex V activity was evaluated with the MitoTox™ Complex V OXPHOS Activity Assay Kit (ab109907). Cellular ATP content was quantified using the ATP Assay Kit (Colorimetric/Fluorometric) (ab83355). Intracellular ROS levels were detected with the Cellular ROS Assay Kit (Deep Red) (ab186029). Cellular estrogen levels were assessed using the Human Estrogen ELISA Kit (ab285239).

### Generation of chemoresistant cell lines

Chemoresistant SW480 cell lines were generated by continuous exposure to increasing concentrations of 5-fluorouracil (5FU), irinotecan (IRI), or oxaliplatin (OXA) over a period of 8 months. The initial concentrations were 1 µM for 5FU, 0.5 µM for IRI, and 0.2 µM for OXA. Drug concentrations were gradually increased every 2–3 passages. The final concentrations used were 8 µM for 5FU, 5 µM for IRI, and 1.5 µM for OXA. Resistant phenotypes were confirmed by dose-response curve analysis comparing parental and resistant cells.

### Lentiviral transduction

The coding sequences of wild-type (WT) CYP19A1 and its mutants, D309N and Y361F, were cloned into the pLenti-CMV-Puro-DEST vector (Addgene, #17452). Lentiviral particles were produced by co-transfecting HEK293T cells with the lentiviral vector and packaging plasmids (pMD2. G and psPAX2) using Lipofectamine 3000. Virus-containing supernatants were collected 48 and 72 h after transfection, filtered through a 0.45 μm filter, and used to infect CYP19A1 knockout SW480 and HT29 cells in the presence of 8 µg/mL polybrene. Transduced cells were selected with 2 µg/mL puromycin for 7 days.

### Dose-response curve analysis

Cell viability was assessed using the sulforhodamine B (SRB) assay. Cells were seeded in 96-well plates at a density of 3,000 cells per well and allowed to attach overnight. The cells were then treated with increasing concentrations of chemotherapy drugs (5-fluorouracil, irinotecan, or oxaliplatin) for 72 h. Following treatment, cells were fixed with 10% trichloroacetic acid (TCA) for 1 h at 4 °C, washed with water, and stained with 0.4% SRB solution for 30 min at room temperature. The excess dye was removed by washing with 1% acetic acid, and the bound dye was solubilized with 10 mM Tris base. The absorbance was measured at 565 nm using a microplate reader. Dose-response curves were generated using the Four Parameter Logistic (4PL) model.

### Mitochondrial isolation and transfer

Mitochondria were isolated from wild-type SW480 cells using the Mitochondria Isolation Kit for Cultured Cells (Thermo Fisher Scientific) according to the manufacturer’s instructions. The isolated mitochondria were quantified using the Pierce BCA Protein Assay Kit. For mitochondrial transfer, chemoresistant CYP19A1 knockout cells were seeded in 6-well plates and cultured until 60–80% confluent. Isolated mitochondria (100 µg protein/well) were directly added to the culture medium of chemoresistant CYP19A1 knockout cells in each well. Cells were then incubated with the isolated mitochondria for 24 h at 37 °C in a 5% CO2 incubator. After incubation, cells were harvested for subsequent analyses.

### Statistical analysis

Data are presented as mean ± standard deviation (SD). Statistical significance was determined using Student’s t-test for pairwise comparisons. P-values less than 0.05 were considered statistically significant and indicated as follows: * *P* < 0.05, ** *P* < 0.01, *** *P* < 0.001, ns (not significant) *P* > 0.05.

## Results

### CYP19A1 is overexpressed and localizes to mitochondria in colorectal cancer cells

**Fig. 1:**
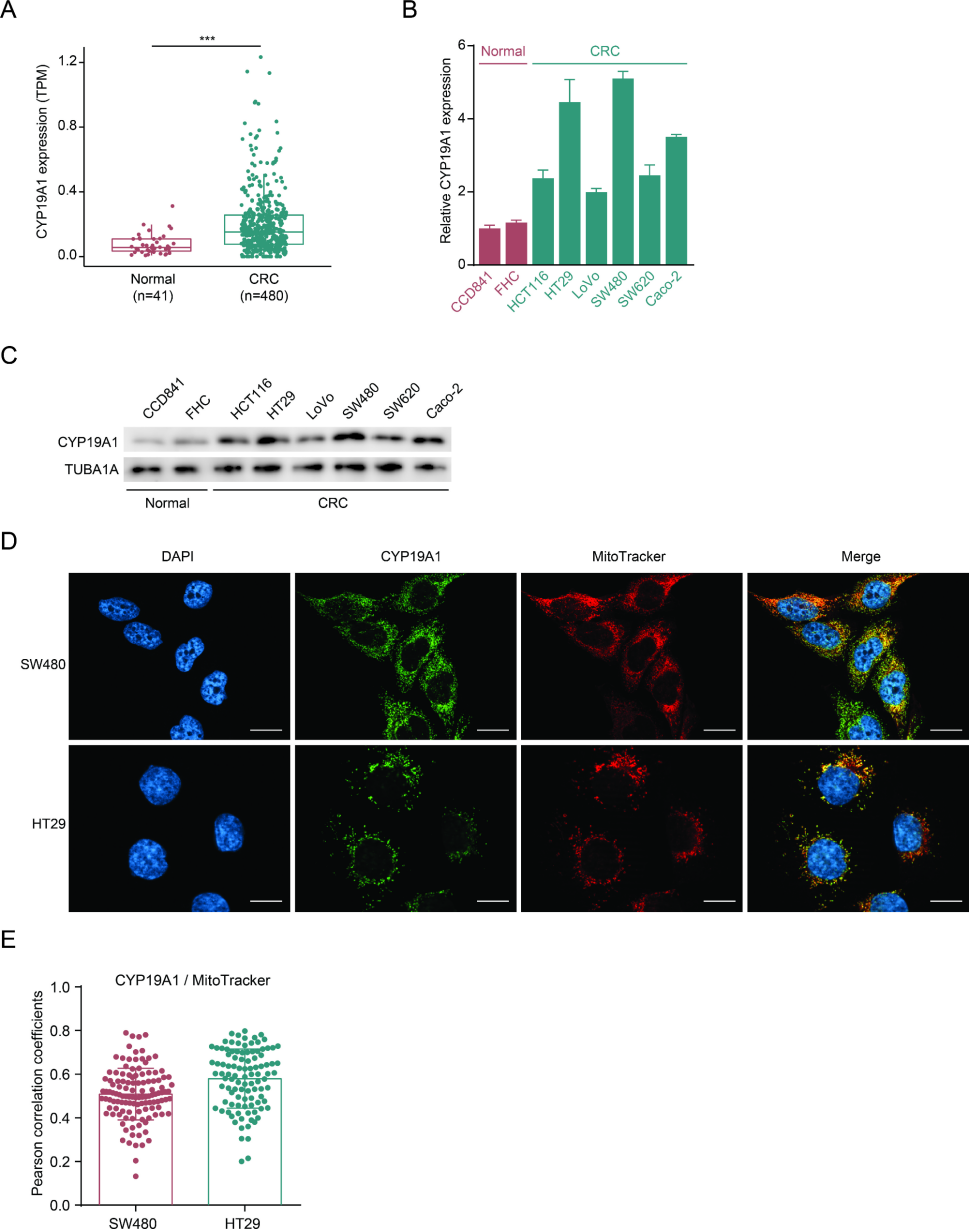
CYP19A1 expression and localization in normal and CRC tissues and cells **(A)** CYP19A1 mRNA expression levels in normal colon tissues and CRC tissues from The Cancer Genome Atlas (TCGA) COAD dataset. The data were analyzed using Wilcoxon rank-sum test (*** *p* < 0.001). **(B)** Relative CYP19A1 mRNA levels in various normal colon and CRC cell lines were determined by real-time PCR (*n* = 3). The expression levels were normalized to the CCD841 normal colon cell line. Data are presented as mean ± SD. **(C)** Western blot analysis of CYP19A1 and TUBA1A (loading control) protein levels in normal colon and CRC cell lines. **(D)** Representative immunofluorescence images showing staining of DAPI (blue, nuclear stain), CYP19A1 (green), MitoTracker (red, mitochondrial stain), and their merged images in SW480 and HT29 CRC cell lines. The colocalization of CYP19A1 and mitochondria is shown in yellow or orange in the merged images. Scale bar: 20 μm. **(E)** Quantitative analysis of CYP19A1 and MitoTracker colocalization from the immunofluorescence images in **(D)**, presented as Pearson correlation coefficients for SW480 (*n* = 121) and HT29 (*n* = 102) CRC cell lines. Each dot represents the correlation coefficient for an individual cell. The bar graphs show the mean Pearson correlation coefficient, with error bars representing standard deviation (SD)

To investigate the role of CYP19A1 in CRC, we first examined its mRNA expression levels in normal colon tissues and CRC tissues using data from The Cancer Genome Atlas (TCGA) dataset. We found that CYP19A1 mRNA levels were significantly higher in CRC tissues compared to normal colon tissues (Fig. 1A). We further assessed CYP19A1 mRNA expression in various normal colon and CRC cell lines using real-time PCR. Consistent with the TCGA data, CYP19A1 mRNA levels were elevated in all CRC cell lines compared to the normal colon cell line CCD841 (Fig. 1B). Western blot analysis confirmed the increased CYP19A1 protein levels in CRC cell lines (Fig. 1C). Taken together, these results demonstrate that CYP19A1 is overexpressed at both mRNA and protein levels in CRC tissues and cell lines, suggesting its potential involvement in CRC.

To gain further insights into CYP19A1’s potential functions, we sought to determine its subcellular localization. While publicly available data from the Human Protein Atlas database revealed that CYP19A1 localizes to mitochondria in various cell types [22], the mitochondrial localization of CYP19A1 in CRC cells has not been previously reported. To address this, we performed immunofluorescence staining of CYP19A1 in SW480 and HT29 CRC cells. Strikingly, the fluorescence signal of CYP19A1 exhibited a clear colocalization with the mitochondrial marker MitoTracker in both cell lines (Fig. 1D), confirming that CYP19A1 indeed resides in the mitochondria of CRC cells. This mitochondrial localization of CYP19A1 suggests that it may play a role in regulating mitochondrial functions in CRC cells.

Additionally, previous studies have indicated that CYP19A1 can also localize to the endoplasmic reticulum [23, 24]. To examine whether this localization pattern is also present in CRC cells, we performed immunofluorescence staining using calreticulin as an endoplasmic reticulum marker. Our results demonstrate that a portion of CYP19A1 indeed colocalizes with calreticulin in both SW480 and HT29 cell lines (Figure S1), confirming that CYP19A1 is also present in the endoplasmic reticulum of CRC cells. This dual localization of CYP19A1 to both mitochondria and the endoplasmic reticulum suggests that it may have diverse functions in different cellular compartments in CRC cells.

### CYP19A1 loss impairs mitochondrial respiration in CRC cells

**Fig. 2:**
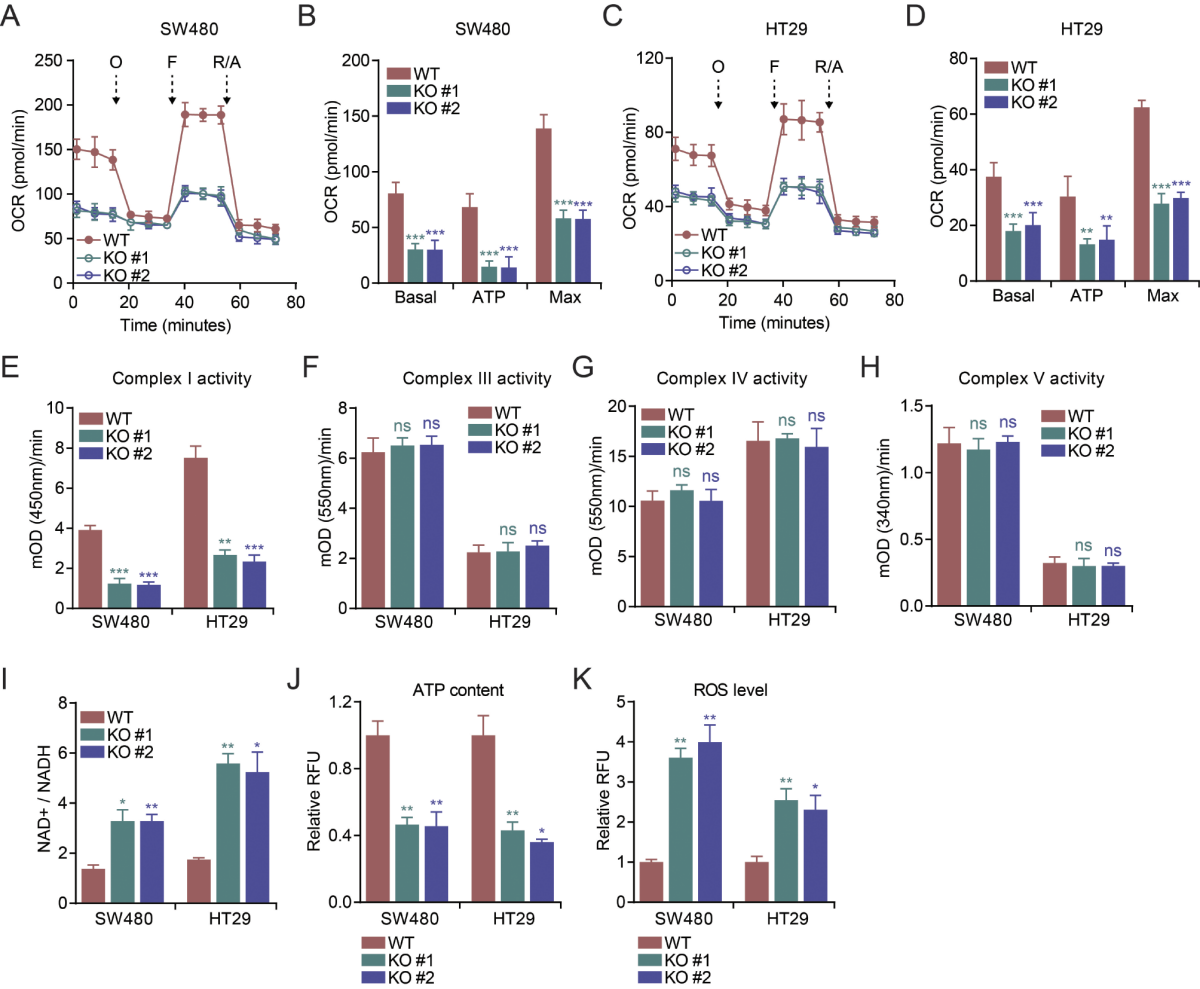
Loss of CYP19A1 inhibits mitochondrial respiration function in CRC cells. CYP19A1 was knocked out using CRISPR-Cas9 technology in SW480 and HT29 CRC cell lines, and two different knockout clones (KO #1 and KO #2) were selected for each cell line. **(A-D)** Oxygen consumption rate (OCR) of wild-type (WT) and CYP19A1 knockout SW480 and HT29 cells was measured using the Seahorse Mito Stress Test. Cells were sequentially treated with oligomycin (O), FCCP (F), and rotenone/antimycin A (R/A) to assess basal, ATP-linked, and maximal respiration. **(E-K)** Mitochondrial complex I (E), III (F), IV (G), and V (H) activities, NAD+/NADH ratio **(I)**, cellular ATP content **(J)**, and reactive oxygen species (ROS) levels **(K)** in WT or CYP19A1 knockout SW480 and HT29 cells were measured using commercially available assay kits. Statistical significance of KO groups compared to the WT group was determined using Student’s t-test (* *p* < 0.05, ** *p* < 0.01, *** *p* < 0.001, ns: not significant). Data are presented as mean ± SD. **(A-D)*** n* = 6; **(E-K)*** n* = 3

To investigate the functional impact of CYP19A1 on mitochondrial respiration, we generated CYP19A1 knockout SW480 and HT29 cell lines using CRISPR-Cas9 technology. Two independent knockout clones (KO #1 and KO #2) were selected for each cell line (Figure S2A). Cell proliferation assays showed no significant difference between wild-type and CYP19A1 knockout cells (Figure S2B, S2C). However, when we investigated mitochondrial respiration using the Seahorse Mito Stress Test, we found that CYP19A1 knockout significantly reduced basal, ATP-linked, and maximal respiration in CRC cells (Fig. 2A-D) cells compared to their wild-type (WT) counterparts. Interestingly, while CYP19A1 knockout led to a significant decrease in mitochondrial complex I activity (Fig. 2E), the activities of complex III, IV, and V remained unaffected (Fig. 2F-H). Moreover, CYP19A1 knockout cells exhibited reduced NAD+/NADH ratio (Fig. 2I) and ATP content (Fig. 2J), and increased reactive oxygen species (ROS) levels (Fig. 2K). These findings suggest that CYP19A1 is crucial for maintaining complex I activity and optimal mitochondrial respiration in CRC cells, and its loss leads to a metabolic imbalance characterized by reduced energy production and increased oxidative stress.

### CYP19A1 regulates mitochondrial function through its enzymatic activity and estrogen biosynthesis

**Fig. 3:**
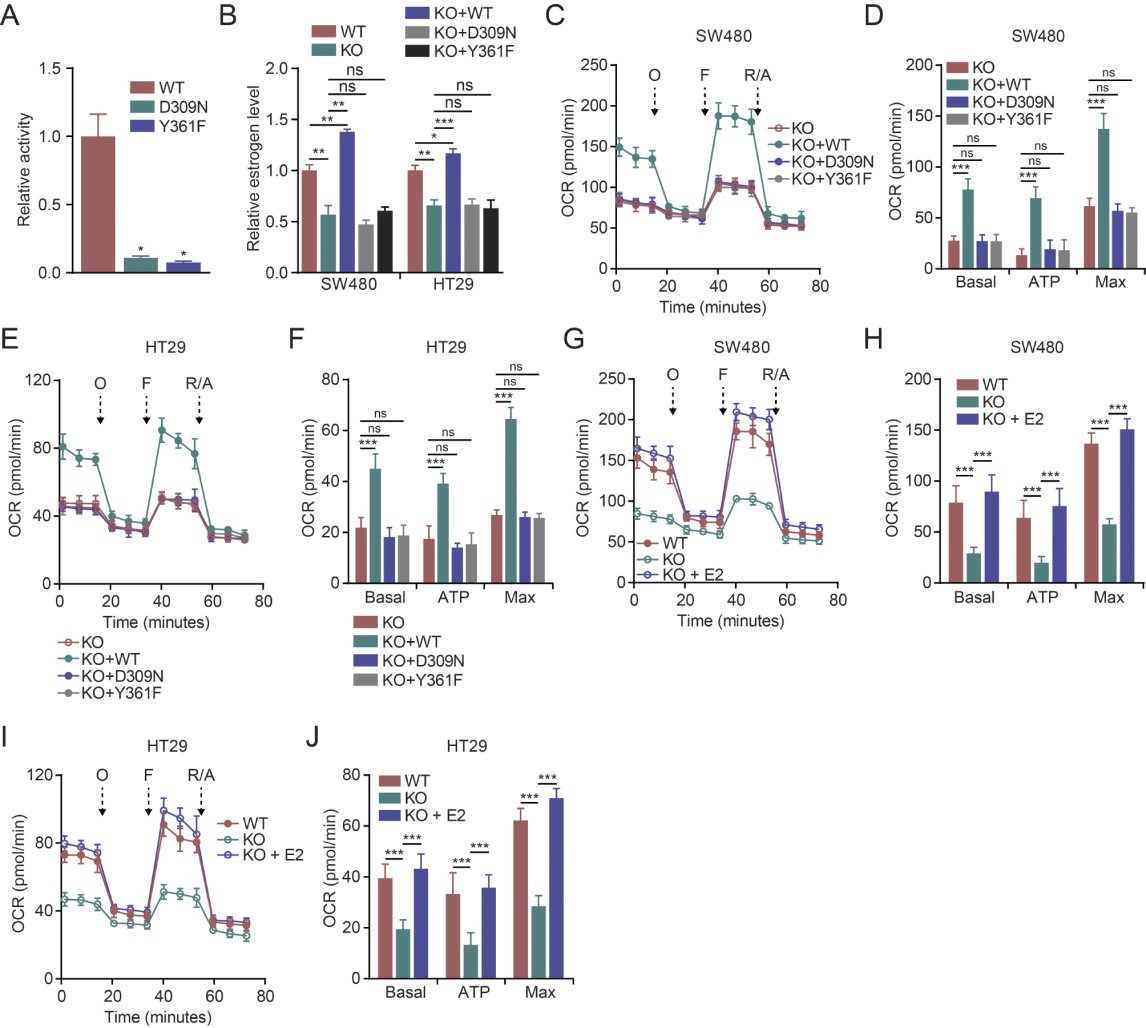
CYP19A1 regulates mitochondrial function depending on its enzymatic activity and estrogen biosynthesis. **(A)** The in vitro enzymatic activity of wild-type (WT) CYP19A1 and its mutants, D309N and Y361F, was determined using an activity assay. **(B-F)** CYP19A1 knockout SW480 and HT29 cells were transduced with lentiviral vectors to express exogenous WT, D309N, or Y361F CYP19A1, generating four cell lines: KO, KO + WT, KO + D309N, and KO + Y361F. **(B)** Relative estrogen levels were measured in WT, KO, KO + WT, KO + D309N, and KO + Y361F cells using a commercially available assay kit. **(C-F)** The OCR of KO, KO + WT, KO + D309N, and KO + Y361F cells was measured using the Seahorse Mito Stress Test. Basal, ATP-linked, and maximal respiration were assessed by sequential treatment with oligomycin (O), FCCP (F), and rotenone/antimycin A (R/A). **(G-J)** The OCR of wild-type and CYP19A1 knockout SW480 and HT29 cells with or without estradiol (E2) treatment was measured using the Seahorse Mito Stress Test. Statistical significance was determined using Student’s t-test according to the indicated comparisons in each panel (* *p* < 0.05, ** *p* < 0.01, *** *p* < 0.001, ns: not significant). Data are presented as mean ± SD. (**A, B**) *n* = 3; **(C-J)*** n* = 6

CYP19A1 is well-known for its role in estrogen biosynthesis, catalyzing the conversion of androgens to estrogens [14]. Previous study has shown that the D309N and Y361F mutations can render CYP19A1 catalytically inactive [25]. To confirm this, we performed an in vitro enzymatic activity assay on wild-type (WT) CYP19A1 and its D309N and Y361F mutants. The assay verified the loss of function in the D309N and Y361F mutants (Fig. 3A), consistent with the earlier report [25]. Thus, to determine whether the enzymatic activity of CYP19A1 is required for its role in mitochondrial function, we used lentiviral vectors to exogenously express WT CYP19A1, D309N, or Y361F in CYP19A1 knockout cells, generating stable CRC cell lines expressing these proteins. As expected, the expression of WT CYP19A1, but not the catalytically inactive mutants, elevated estrogen levels in CYP19A1 knockout cells (Fig. 3B). Interestingly, the exogenous expression of WT CYP19A1 in CYP19A1 knockout cells resulted in significantly higher estrogen levels compared to wild-type cells (Fig. 3B). This observation suggests that cells expressing endogenous CYP19A1 may not be operating at their maximum capacity for estrogen synthesis, indicating potential for increased estrogen production by overexpression of CYP19A1. Next, we investigated the impact of CYP19A1 enzymatic activity on mitochondrial respiration. The Seahorse Mito Stress Test demonstrated that the expression of WT CYP19A1, but not the D309N or Y361F mutants, rescued the impaired mitochondrial respiration in CYP19A1 knockout CRC cells (Fig. 3C-F). Moreover, estradiol supplementation completely restored mitochondrial respiration in CYP19A1 knockout cells (Fig. 3G-J), suggesting that CYP19A1 regulates mitochondrial function through its enzymatic activity and estrogen biosynthesis.

### Pharmacological inhibition of CYP19A1 or mitochondrial complex I suppresses mitochondrial respiration in CRC cells

**Fig. 4:**
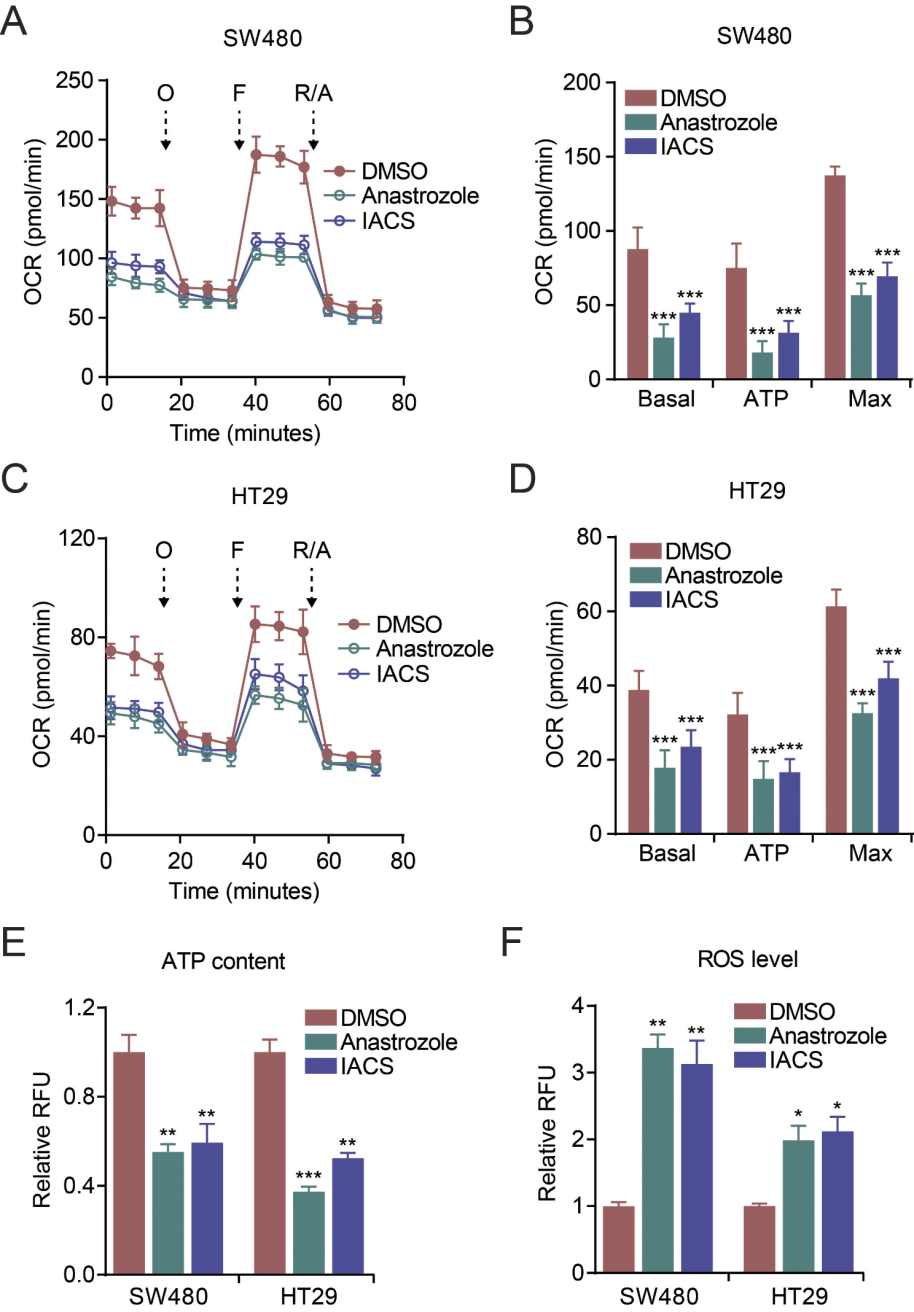
Inhibition of CYP19A1 or mitochondrial complex I suppresses mitochondrial respiration in CRC cells. **(A-D)** SW480 and HT29 cells were treated with DMSO (vehicle control), anastrozole (CYP19A1 inhibitor), or IACS-010759 (IACS, mitochondrial complex I inhibitor), and the OCR was measured using the Seahorse Mito Stress Test. Basal, ATP-linked, and maximal respiration were assessed by sequential treatment with oligomycin (O), FCCP (F), and rotenone/antimycin A (R/A). **(E, F)** Relative cellular ATP content (E) and ROS levels (F) in SW480 and HT29 cells treated with DMSO, anastrozole, or IACS were measured using commercially available assay kits. Statistical significance of the anastrozole and IACS groups compared to the DMSO group was determined using Student’s t-test (* *p* < 0.05, ** *p* < 0.01, *** *p* < 0.001). Data are presented as mean ± SD. **(A-D)*** n* = 6; **(E, F)*** n* = 3

To further validate the role of CYP19A1 in mitochondrial function, we next sought to determine the effects of pharmacological inhibition of CYP19A1 and mitochondrial complex I on mitochondrial respiration in CRC cells. We treated CRC cells with anastrozole, a specific CYP19A1 inhibitor [26–28], or IACS-010759 (IACS), a mitochondrial complex I inhibitor [29, 30]. Consistent with our findings in CYP19A1 knockout cells, both anastrozole and IACS significantly suppressed mitochondrial respiration in CRC cells (Fig. 4A-D). Furthermore, anastrozole and IACS treatment reduced cellular ATP content (Fig. 4E) and increased ROS levels (Fig. 4F) in CRC cells. These results provide additional evidence supporting the critical role of CYP19A1 and mitochondrial complex I in regulating mitochondrial respiration and cellular energy homeostasis in CRC cells.

### Targeting CYP19A1 reverses chemoresistance in CRC cells by regulating mitochondrial function and complex I activity

**Fig. 5:**
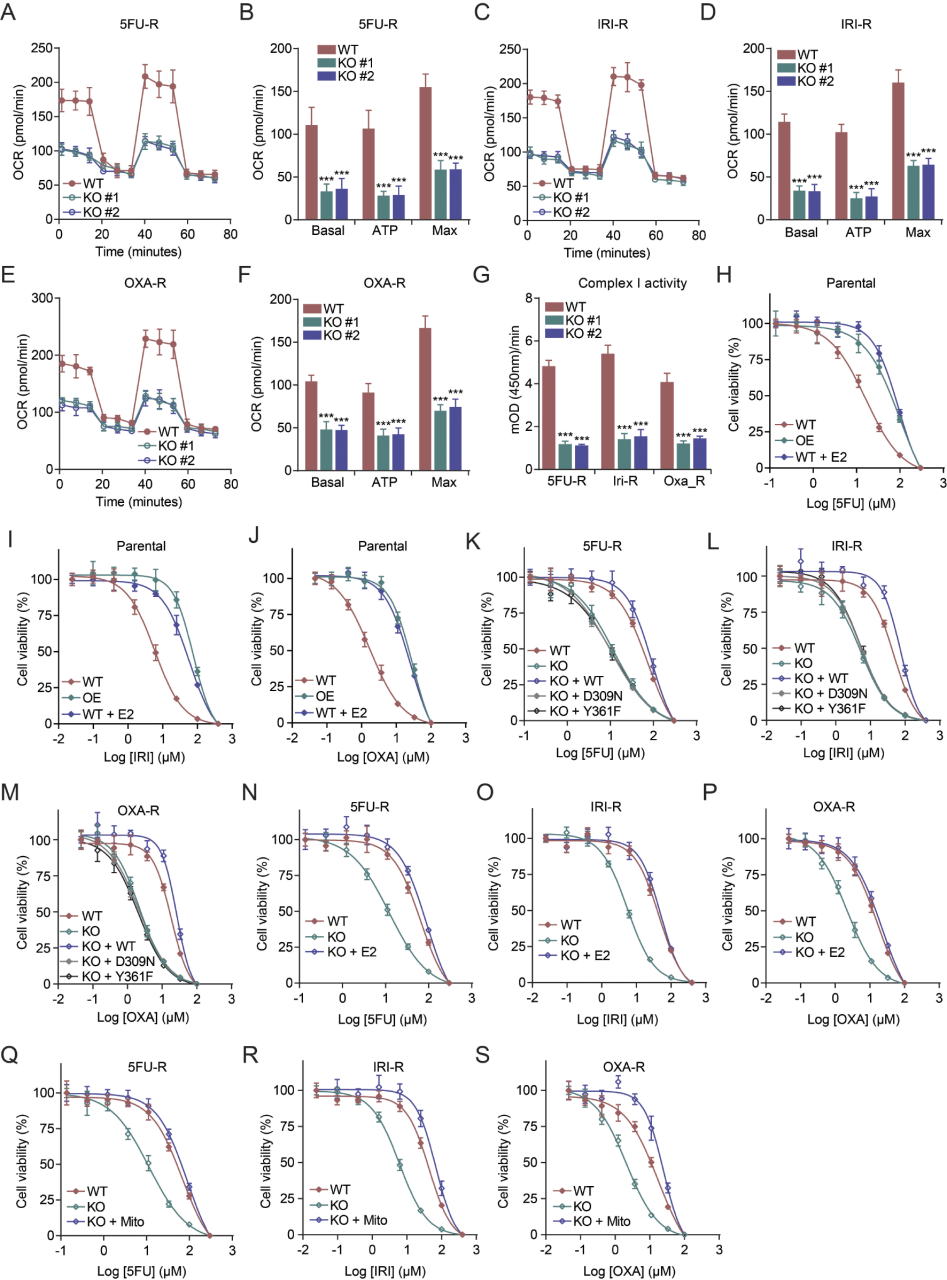
CYP19A1 knockout reverses chemoresistance in CRC cells. **(A-F)** The OCR of wild-type and CYP19A1 knockout chemoresistant SW480 cells was measured using the Seahorse Mito Stress Test. Basal, ATP-linked, and maximal respiration were assessed by sequential treatment with oligomycin (O), FCCP (F), and rotenone/antimycin A (R/A) in 5-fluorouracil (5FU-R; A, B), irinotecan (IRI-R; C, D), and oxaliplatin (OXA-R; E, F) resistant cells. **(G)** Mitochondrial complex I activity in 5FU-R, IRI-R, and OXA-R cells was measured using a commercially available assay kit. **(H-J)** Dose-response curves of parental SW480 cells treated with 5FU, IRI, and OXA in wild-type (WT), CYP19A1 overexpressing (OE), and estradiol-treated (WT + E2) conditions. **(K-M)** Dose-response curves showing cell viability of 5FU, IRI, and OXA in 5FU-R, IRI-R, and OXA-R cell lines treated with different concentrations of 5FU, IRI, and OXA, respectively. Curves are shown for WT, knockout (KO), and KO cells complemented with constructs expressing WT or mutant (D309N, Y361F) CYP19A1. **(N-P)** Dose-response curves illustrating cell viability in 5FU-R, IRI-R, and OXA-R lines exposed to varying concentrations of their respective drugs in WT, KO, and KO cells treated with estradiol (KO + E2). **(Q-S)** Dose-response curves demonstrating cell viability in 5FU-R, IRI-R, and OXA-R lines exposed to varying concentrations of their respective drugs in WT, KO, and KO cells supplemented with mitochondria extracted from WT cells (KO + Mito). Statistical significance of KO groups compared to the WT group was determined using Student’s t-test (*** *p* < 0.001). Data are presented as mean ± SD. **(A-F)*** n* = 6; **(G-S)*** n* = 3

Previous studies have shown that mitochondrial function mediates chemoresistance in CRC [31, 32]. Thus, we hypothesized that targeting CYP19A1 and its downstream effector complex I could potentially reverse chemoresistance. To test this hypothesis, we first established 5FU-resistant (5FU-R), oxaliplatin-resistant (OXA-R), and irinotecan-resistant (IRI-R) CRC cell lines, and verified their resistant phenotypes (Figure S3). Having confirmed the successful establishment of chemoresistant cell lines, we next examined the impact of CYP19A1 knockout on mitochondrial respiration and complex I activity in these resistant cells. Notably, CYP19A1 knockout significantly suppressed mitochondrial respiration (Fig. 5A-F) and reduced complex I activity in chemoresistant CRC cells (Fig. 5G), effectively reversing their chemoresistance (Figure S4).

To further understand the role of CYP19A1 in chemoresistance development, we investigated its effects in the parental CRC cells. Interestingly, we observed that overexpression of CYP19A1 or treatment with estradiol increased the tolerance of these parental cells to chemotherapeutic drugs (Fig. 5H-J). This finding suggested a possible link between CYP19A1, estrogen signaling, and the development of chemoresistance.

To further investigate whether CYP19A1 relies on its enzymatic activity to influence acquired chemoresistance, we exogenously introduced wild-type and catalytically inactive mutant forms of CYP19A1 into CYP19A1 knockout cells. We found that the introduction of wild-type CYP19A1 could reverse the chemosensitization mediated by CYP19A1 deficiency (Fig. 5K-M), while the catalytically inactive mutant CYP19A1 could not (Fig. 5K-M). Similarly, we observed that exogenous addition of estradiol could also reverse the chemosensitization caused by CYP19A1 deficiency (Fig. 5N-P). These results indicate that CYP19A1-mediated estrogen synthesis is crucial in regulating acquired resistance of CRC cells to chemotherapeutic drugs.

To further confirm that CYP19A1/estrogen axis modulates chemoresistance through regulating mitochondrial function, we performed a mitochondrial rescue experiment. We isolated mitochondria from WT cells and introduced them into CYP19A1 knockout resistant cells. Strikingly, exogenous supplementation of WT mitochondria successfully rescued the chemoresistance reversal effect of CYP19A1 knockout (Fig. 5Q-S), indicating that CYP19A1 influences chemoresistance by suppressing mitochondrial function.

**Fig. 6:**
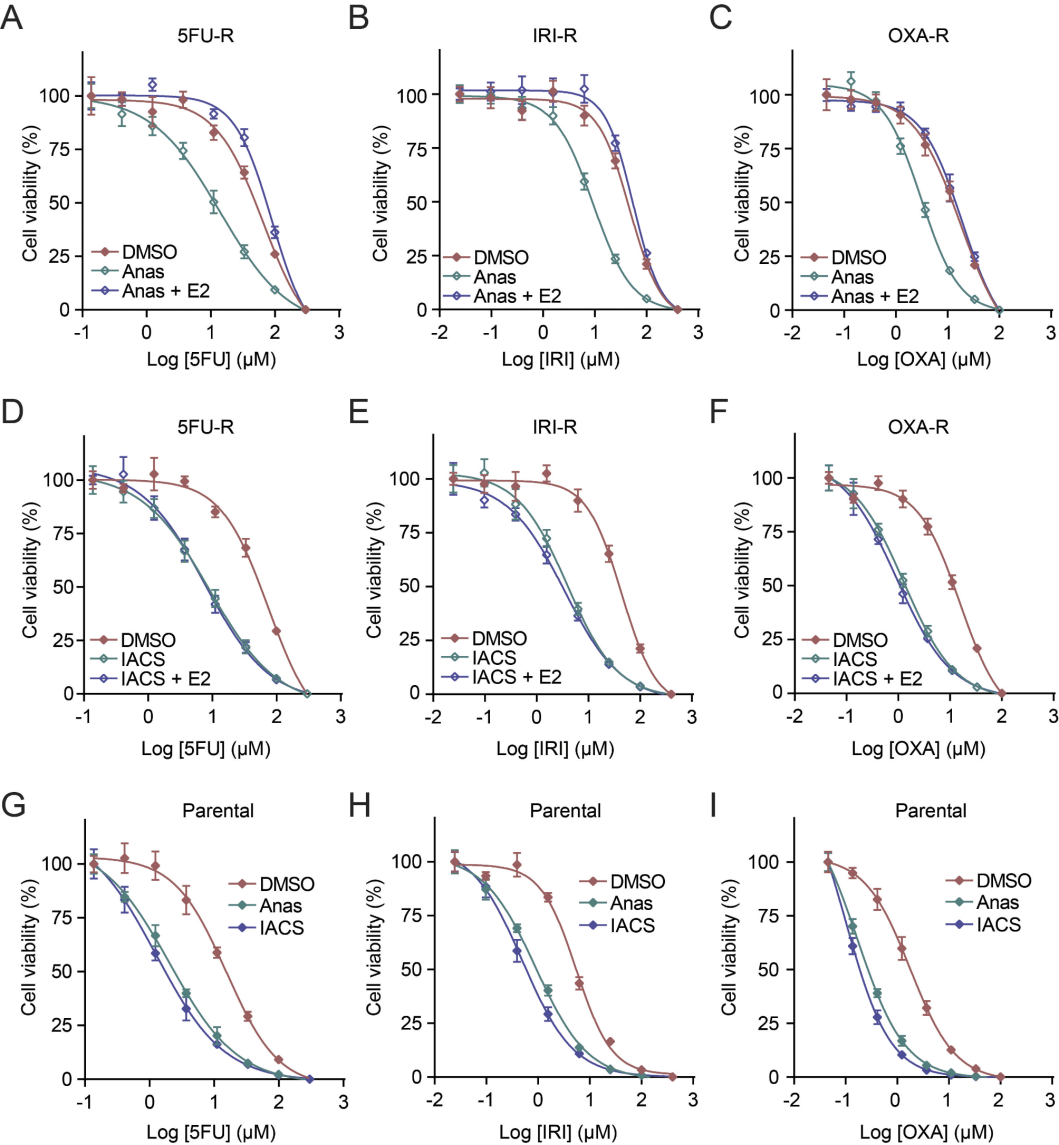
Estrogen supplementation reverses the chemosensitizing effects of CYP19A1 inhibitors but not mitochondrial complex I inhibitors in CRC cells. **(A-F)** Cell viability of 5-fluorouracil (5FU-R; A, D), irinotecan (IRI-R; B, E), and oxaliplatin (OXA-R; C, F) resistant SW480 cells treated with DMSO (vehicle control), anastrozole (Anas, CYP19A1 inhibitor), anastrozole plus estradiol (Anas + E2), IACS-010759 (IACS, mitochondrial complex I inhibitor), or IACS-010759 plus estradiol (IACS + E2) was assessed using the sulforhodamine B (SRB) assay. Dose-response curves were generated using the Four Parameter Logistic (4PL) model. **(G-I)** Dose-response curves of parental SW480 cells treated with varying concentrations of 5FU (G), IRI (H), and OXA (I) in combination with DMSO, Anas, or IACS. Data points represent mean ± SD. **(A-I)*** n* = 3

Building upon these findings, we then treated chemoresistant CRC cells with anastrozole or IACS to further validate the role of CYP19A1 and complex I in chemoresistance regulation. Consistent with our findings in CYP19A1 knockout cells, pharmacological inhibition of either CYP19A1 by anastrozole or complex I by IACS significantly sensitized 5FU-R (Fig. 6A, D), IRI-R (Fig. 6B, E), and OXA-R (Fig. 6C, F) cells to their respective chemotherapy drugs, confirming that targeting CYP19A1 or complex I can effectively reverse chemoresistance. Importantly, while estradiol supplementation completely abrogated the sensitization effect of anastrozole (Fig. 6A-C), it failed to rescue the chemosensitization induced by IACS (Fig. 6D-F). These results suggest that CYP19A1 regulates chemoresistance through a CYP19A1/estrogen/complex I axis, with complex I being a key downstream effector of CYP19A1 and estrogen signaling.

To further enhance the clinical implication of our findings, we also investigated the effects of anastrozole and IACS on chemosensitivity in parental CRC cells. Consistent with our observations in chemoresistant cells, we observed that treatment with either anastrozole or IACS significantly increased the sensitivity of parental cells to chemotherapeutic agents (Fig. 6G-I). These results demonstrate that inhibition of CYP19A1 or complex I can effectively sensitize both chemoresistant and chemosensitive CRC cells to standard chemotherapeutic drugs, underscoring the potential broad applicability of targeting this pathway in colorectal cancer treatment.

### High CYP19A1 expression correlates with poor overall survival in chemotherapy-treated CRC patients

**Fig. 7:**
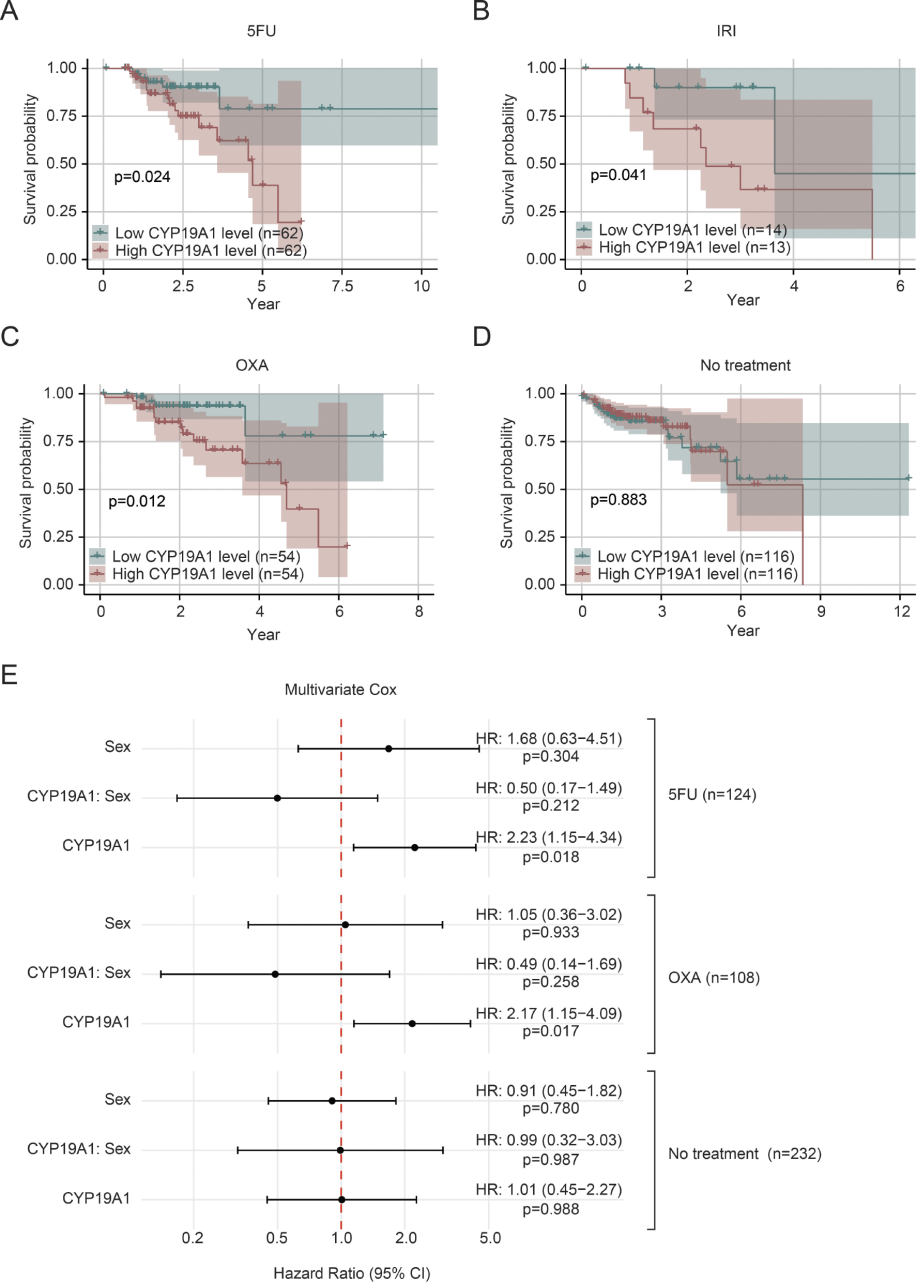
High CYP19A1 expression is associated with poor prognosis in CRC patients receiving chemotherapy but not in untreated patients. **(A-D)** Kaplan-Meier survival curves of CRC patients treated with 5-fluorouracil (5FU; A), irinotecan (IRI; B), oxaliplatin (OXA; C), or no chemotherapy (D), stratified by CYP19A1 expression level (low vs. high). The p-values were calculated using the log-rank test. A p-value less than 0.05 was considered statistically significant. (E) Forest plot showing the impact of CYP19A1 expression and patient sex on chemoresistance across different treatment groups based on multivariate Cox regression analyses. Hazard ratios (HR) with 95% confidence intervals (CI) and P-values are presented for CYP19A1 expression, sex, and their interaction in 5FU, OXA, and no chemotherapy treatment group. The vertical dashed line represents HR = 1. The IRI group was excluded from this analysis due to its small sample size (*n* = 27) and low event count (10 events), which led to unstable estimates and potential overfitting in the statistical model. This limitation prevented reliable conclusions for the IRI group, necessitating its omission from the final comparative analysis

To further validate the clinical relevance of our findings and explore the potential role of CYP19A1 in modulating chemotherapy response in CRC patients, we analyzed the relationship between CYP19A1 expression and patient survival using data from TCGA database. Kaplan-Meier survival analysis revealed that high CYP19A1 expression was significantly associated with poorer overall survival in 5FU (Fig. 7A), IRI (Fig. 7B), and OXA (Fig. 7C) treated patients, but not in untreated patients (Fig. 7D). These findings highlight the clinical significance of CYP19A1 in CRC and its potential role in modulating chemotherapy response, consistent with our in vitro findings demonstrating that CYP19A1 reverses chemoresistance in CRC cells.

Given the known association between biological sex and estrogen levels, we further investigated whether the patient’s sex (male/female) influences the relationship between CYP19A1 expression and chemoresistance. To this end, we performed multivariate Cox regression analyses for each treatment group, incorporating CYP19A1 expression, sex, and their interaction (Fig. 7E). The results show that in the 5FU-treated group, high CYP19A1 expression was significantly associated with increased risk of death (HR = 2.23, 95% CI: 1.15–4.34, *p* = 0.018), while neither sex (HR = 1.68, 95% CI: 0.63–4.51, *p* = 0.304) nor the interaction between CYP19A1 and sex (HR = 0.50, 95% CI: 0.17–1.49, *p* = 0.212) showed significant effects (Fig. 7E). Similar trends were observed in the oxaliplatin (OXA) group, where high CYP19A1 expression was also significantly associated with increased death risk (HR = 2.17, 95% CI: 1.15–4.09, *p* = 0.017), while sex (HR = 1.05, 95% CI: 0.36–3.02, *p* = 0.933) and the CYP19A1-sex interaction (HR = 0.49, 95% CI: 0.14–1.69, *p* = 0.258) remained non-significant (Fig. 7E). Consistent with the Kaplan-Meier analysis, the Cox regression for the untreated group showed no significant associations for CYP19A1 expression (HR = 1.01, 95% CI: 0.45–2.27, *p* = 0.988), sex (HR = 0.91, 95% CI: 0.45–1.82, *p* = 0.780), or their interaction (HR = 0.99, 95% CI: 0.32–3.03, *p* = 0.987) (Fig. 7E).

These findings highlight the clinical significance of CYP19A1 in CRC and its potential role in modulating chemotherapy response. The consistent association between high CYP19A1 expression and poor survival outcomes in chemotherapy-treated patients aligns with our in vitro findings demonstrating that CYP19A1 influences chemoresistance in CRC cells. Importantly, while CYP19A1 expression is a significant predictor of survival in chemotherapy, this association does not appear to be significantly modulated by patient sex. The lack of significant association in untreated patients further supports the specific role of CYP19A1 in chemotherapy response rather than in overall disease progression.

In summary, our results demonstrate that CYP19A1 plays a critical role in regulating chemoresistance in CRC cells by modulating mitochondrial function and complex I activity. Mechanistically, CYP19A1 exerts its effects through a CYP19A1/estrogen/complex I signaling axis. Importantly, targeting CYP19A1 or complex I with specific inhibitors effectively reverses chemoresistance in CRC cells, highlighting the therapeutic potential of this approach. Moreover, clinical data from the TCGA database reveals a significant correlation between high CYP19A1 expression and poor overall survival in chemotherapy-treated CRC patients, further substantiating the clinical relevance of our findings. Collectively, our study provides novel insights into the molecular mechanisms underlying chemoresistance in CRC and identifies the CYP19A1/estrogen/complex I axis as a promising therapeutic target for overcoming chemoresistance and improving patient outcomes.

## Discussion

The development of chemoresistance remains a major obstacle in the effective treatment of CRC, leading to poor patient prognosis and increased mortality rates. In this study, we aimed to elucidate the molecular mechanisms underlying chemoresistance in CRC and identify novel therapeutic targets. Our findings reveal a previously unrecognized role for CYP19A1 in regulating chemoresistance through modulation of mitochondrial function and complex I activity, providing new insights into the complex interplay between hormone signaling, mitochondrial metabolism, and drug resistance in CRC. Mitochondria have emerged as key players in cancer progression and therapy response, with increasing evidences suggesting that alterations in mitochondrial metabolism can contribute to the development of drug resistance [31, 32]. Our findings provide a new perspective on the role of mitochondria in CRC chemoresistance, suggesting that targeting the CYP19A1/estrogen/complex I axis could be a promising strategy to overcome drug resistance and enhance treatment efficacy.

The identification of estrogen signaling as a mediator of CYP19A1’s effects on mitochondrial function and chemoresistance adds another layer of complexity to the regulatory network governing therapy response in CRC. While the role of estrogen signaling in CRC remains controversial, our findings suggest that it may play a more significant role than previously appreciated, particularly in the context of chemoresistance. This opens up new avenues for exploring the potential of hormone-based therapies in CRC and highlights the need for further research to fully elucidate the complex interplay between hormone signaling, mitochondrial metabolism, and drug resistance.

The translational relevance of our findings is underscored by the analysis of clinical data from the TCGA database, which revealed a significant correlation between CYP19A1 expression and overall survival in chemotherapy-treated CRC patients. This suggests that CYP19A1 could serve as a valuable biomarker for predicting chemotherapy response and patient prognosis in CRC. However, further validation in larger patient cohorts and prospective clinical trials is needed to confirm its predictive and prognostic value.

While our study provides novel insights into the role of CYP19A1 in CRC chemoresistance, several questions remain unanswered. For instance, the precise molecular mechanisms by which CYP19A1 and estrogen signaling regulate complex I activity and mitochondrial function require further elucidation. Additionally, the potential impact of other hormone signaling pathways, such as androgen signaling, on CRC chemoresistance warrants investigation. Future studies should also focus on exploring the therapeutic potential of targeting the CYP19A1/estrogen/complex I axis in preclinical models of CRC and evaluating the efficacy of combination therapies involving CYP19A1 inhibitors and conventional chemotherapeutic agents.

Another limitation of our study is that the findings are based on in vitro experiments using CRC cell lines without in vivo validation. While cell line studies provide valuable insights into the molecular mechanisms of chemoresistance, they may not fully recapitulate the complex tumor microenvironment and host-tumor interactions that occur in vivo. Future studies should focus on validating our findings in preclinical animal models of CRC to assess the therapeutic potential of targeting the CYP19A1/estrogen/complex I axis and evaluate the impact of CYP19A1 modulation on tumor growth, metastasis, and chemotherapy response.

In conclusion, our study uncovers a novel role for CYP19A1 in regulating chemoresistance in CRC through modulation of mitochondrial function and complex I activity, and identifies the CYP19A1/estrogen/complex I axis as a potential therapeutic target. These findings contribute to our understanding of the molecular basis of chemoresistance in CRC and pave the way for the development of new strategies to enhance the efficacy of chemotherapy and improve patient outcomes. However, further research, including in vivo validation and clinical studies, is needed to fully elucidate the complex regulatory networks governing therapy response in CRC and translate these findings into clinical practice.

## Electronic supplementary material

Below is the link to the electronic supplementary material.

Supplementary Material 1

Supplementary Material 2

## Data Availability

The TCGA CRC dataset used in this study is publicly available in the TCGA repository under the project ID: TCGA-COAD (https://portal.gdc.cancer.gov/).
