# Supplementary figures and images for: CYP19A1 regulates chemoresistance in colorectal cancer through modulation of estrogen biosynthesis and mitochondrial function
Source: Cancer Metab. 2024 Oct 28;12:33. doi: 10.1186/s40170-024-00360-4 (PMC11520061; doi:10.1186/s40170-024-00360-4)

Figure 1C

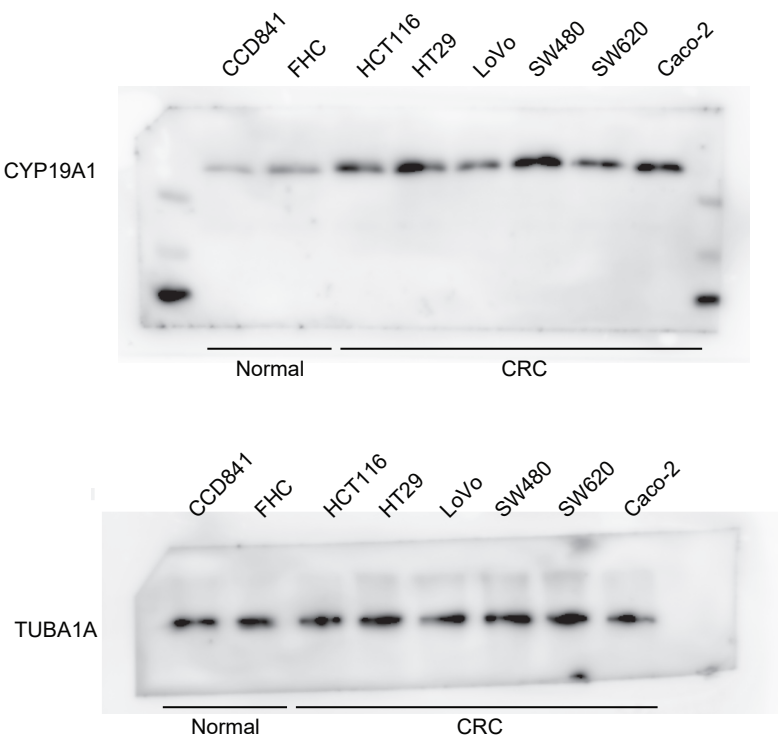

Figure S2

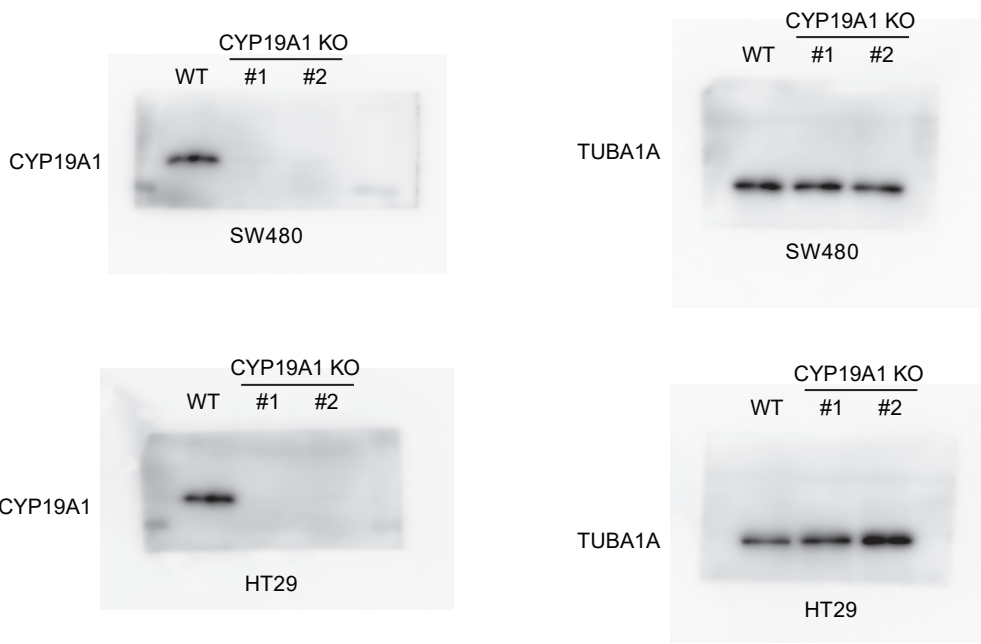

Supplement: Supplementary file 1 — Supplementary Material 1 [file 40170_2024_360_MOESM1_ESM.pdf]
