# Supplementary material for: CYP19A1 regulates chemoresistance in colorectal cancer through modulation of estrogen biosynthesis and mitochondrial function
Source: Cancer Metab. 2024 Oct 28;12:33. doi: 10.1186/s40170-024-00360-4 (PMC11520061; doi:10.1186/s40170-024-00360-4)

## Figure S1. Localization of CYP19A1 to the endoplasmic reticulum in CRC cell lines.

(A) Representative immunofluorescence images showing staining of DAPI (blue, nuclear stain), CYP19A1 (green), calreticulin (red, endoplasmic reticulum marker), and their merged images in SW480 and HT29 cells. The colocalization of CYP19A1 and calreticulin, indicating CYP19A1 localization to the endoplasmic reticulum, is shown in yellow or orange in the merged images. Scale bar: 20 μm. (B) Quantitative analysis of CYP19A1 and calreticulin colocalization from the immunofluorescence images in (A), presented as Pearson correlation coefficients for SW480 (n=112) and HT29 (n=101) cells. Each dot represents the correlation coefficient for an individual cell. The bar graphs show the mean Pearson correlation coefficient, with error bars representing standard deviation (SD). Higher correlation coefficients indicate greater colocalization of CYP19A1 with the endoplasmic reticulum.

## Figure S2. CYP19A1 knockout does not affect proliferation of CRC cells.

(A) Western blot analysis of CYP19A1 expression in wild-type (WT) and two CYP19A1 knockout (KO) clones (#1 and #2) in SW480 and HT29 cell lines. (B, C) Cell proliferation of SW480 (B) and HT29 (C) WT and CYP19A1 KO clones over 5 days, measured by sulforhodamine B (SRB) assay. Data points represent mean ± SD. (B, C) n=3.

## Figure S3. Chemoresistant CRC cells exhibit reduced sensitivity to their respective chemotherapy drugs compared to parental cells.

(A-C) Cell viability of parental and chemoresistant CRC cells treated with increasing concentrations of 5-fluorouracil (5FU; A), irinotecan (IRI; B), or oxaliplatin (OXA; C) was assessed using the sulforhodamine B (SRB) assay. 5FU-R, IRI-R, and OXA-R refer to SW480 cells resistant to 5FU, IRI, and OXA, respectively. Dose-response curves were generated using the Four Parameter Logistic (4PL) model. The reduced sensitivity of chemoresistant cells to their respective drugs confirms the successful establishment of the chemoresistant cell lines. Data are presented as mean ± SD. (A-C) n=3.

## Figure S4. CYP19A1 knockout reverses chemotherapy resistance in CRC cells.

(A-C) Cell viability assays of wild-type (WT) and CYP19A1 knockout (KO #1 and KO #2) chemotherapy-resistant CRC cells was assessed using the sulforhodamine B (SRB) assay. (A) 5-fluorouracil-resistant cells (5FU-R) treated with 5FU, (B) irinotecan-resistant cells (IRI-R) treated with irinotecan, and (C) oxaliplatin-resistant cells (OXA-R) treated with oxaliplatin. Cells were exposed to increasing concentrations of the respective drugs for 72 hours, and cell viability was assessed. Dose-response curves were generated using the Four Parameter Logistic model. Data are presented as mean ± SD. (A-C) n=3.


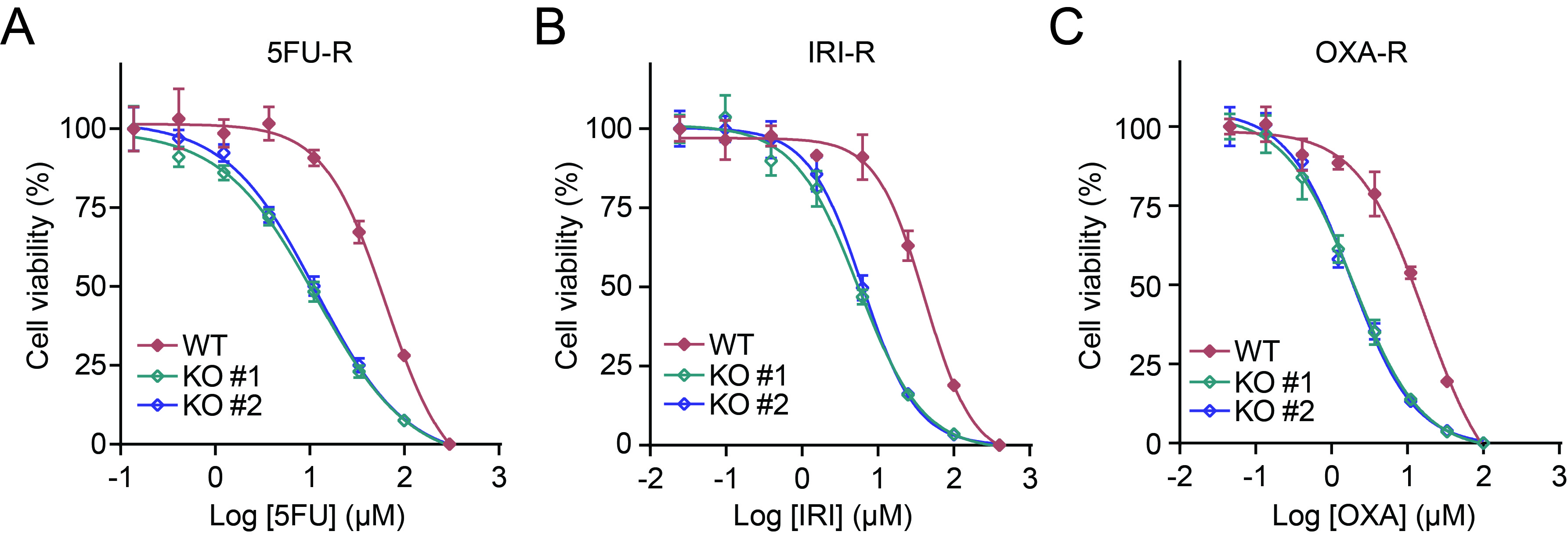

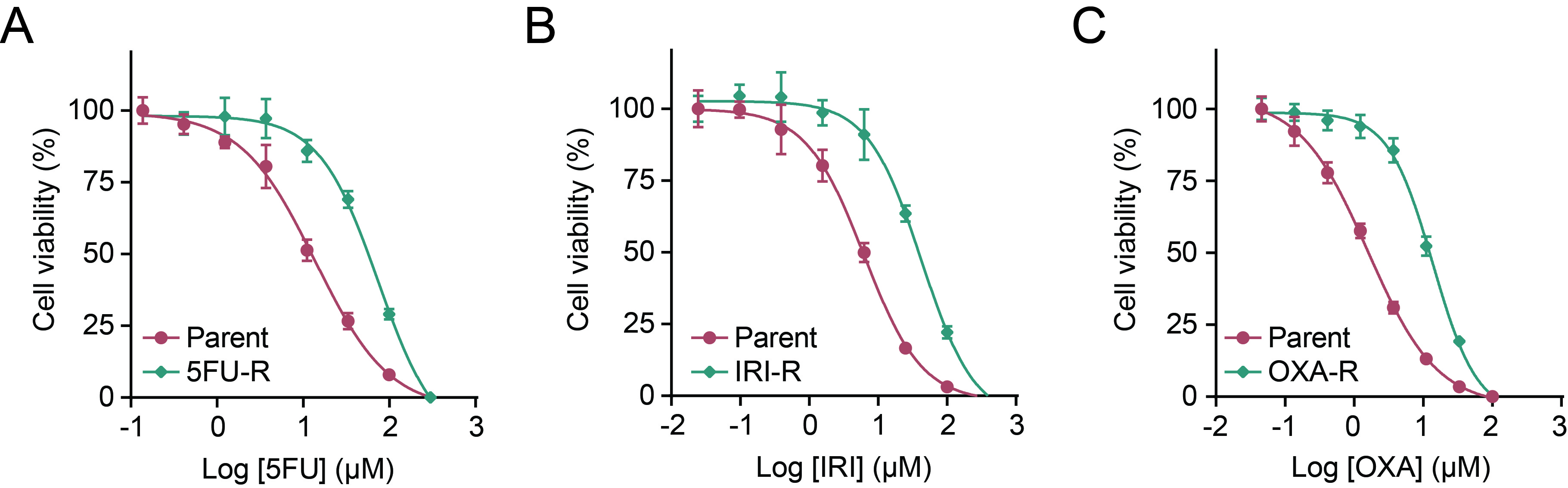

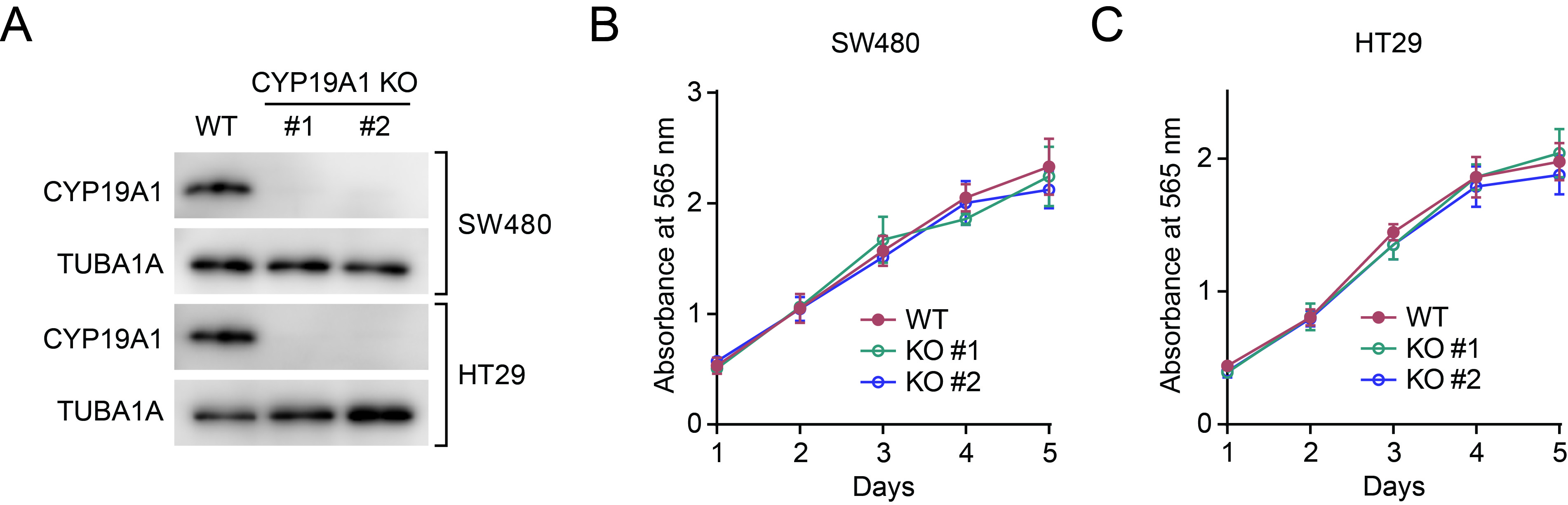

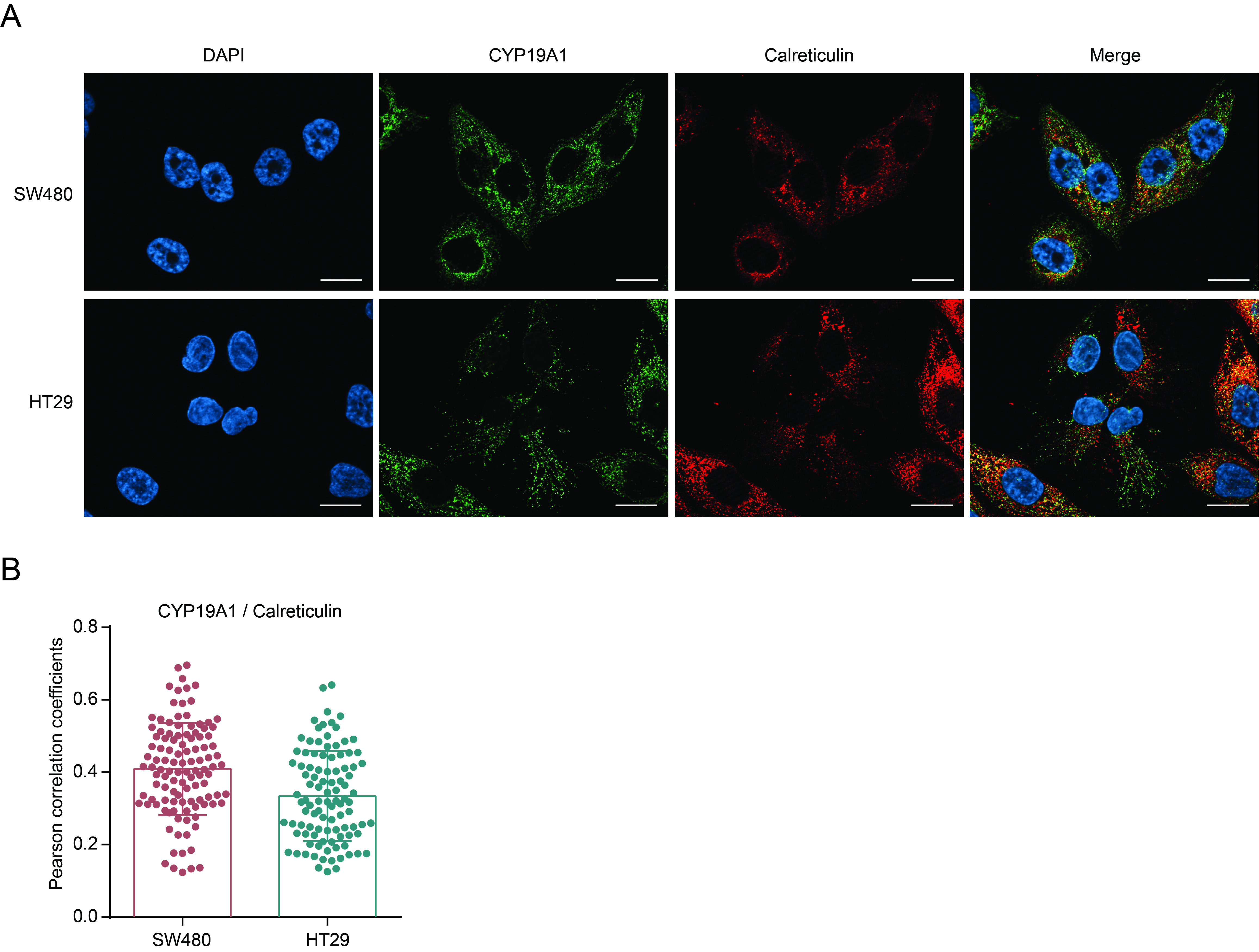

Supplement: Supplementary file 2 — Supplementary Material 2 [file 40170_2024_360_MOESM2_ESM.docx]
